# *Thymus linearis* Extracts Ameliorate Indices of Metabolic Syndrome in Sprague Dawley Rats

**DOI:** 10.1155/2023/5648837

**Published:** 2023-04-21

**Authors:** Yamema Younatan, Muhammad Majid, Abdul Rehman Phull, Muhammad Waleed Baig, Nadeem Irshad, Humaira Fatima, Bakht Nasir, Aroosa Zafar, Abdul Majid, Amna Parveen, Ihsan-ul Haq

**Affiliations:** ^1^Department of Pharmacy, Faculty of Biological Sciences, Quaid-i-Azam University, Islamabad 45320, Pakistan; ^2^Faculty of Pharmacy, Hamdard University, Islamabad 44000, Pakistan; ^3^Department of Food Science and Biotechnology, College of BioNano Technology, Gachon University, Gyeonggi-do, 13120, Republic of Korea; ^4^Department of Biochemistry, Shah Abdul Latif University, Khairpur, 66020 Sindh, Pakistan; ^5^College of Pharmacy, Gachon University, No. 191, Hambakmoero, Yeonsu-gu, Incheon 21936, Republic of Korea

## Abstract

**Materials and Methods:**

The extract library (n-hexane (NH), ethyl acetate (EA), methanol (M), distilled water (DW), and combined extract (CE)) was standardized using *in vitro* phytochemical, antioxidant, and *α*-amylase inhibition assays, after which the protective effect of selected “hit,” i.e., CE against metabolic syndrome, was determined *in vivo*, using rats fed a high-fat diet supplemented with additional cholesterol administration. CE was administered to Sprague Dawley rats in high dose as 100 mg/kg in carboxymethyl cellulose (CMC) (1 ml; 0.75% in DW) and low-dose group as 50 mg/kg in CMC (0.5 ml; 0.75% in DW). After 10 weeks, the effects of CE on insulin resistance, lipid metabolism, nonalcoholic fatty liver disease (NAFLD), oxidative stress, and genotoxicity were assessed through histological, biochemical, and hematological investigations.

**Results:**

Phytochemical analysis including RP-HPLC analysis of the extracts showed that flavonoids and phenolics (myricetin, kaempferol, and apigenin), previously known to be effective against obesity and diabetes, are present in the extracts. Antioxidant studies revealed that the plant possesses a highly significant (*p* < 0.05) concentration of antioxidants. Satisfactory *α*-amylase inhibitory activity was also observed in *in vitro* experiments. *In vivo* studies showed that CE-administered animals had significantly (*p* < 0.05) lower weight gain and smaller adipocytes than the control group. Moreover, CE resisted any significant (*p* < 0.05) change in the organ weights. Analogous to findings from its traditional use, the plant extract had a positive modulatory effect on insulin resistance and hyperglycemia. The study also indicated that CE resisted high-fat diet-induced disturbance in lipid profile and countered any pathological changes in liver enzymes caused by fat-infused diet. Furthermore, a study on endogenous antioxidant levels indicated that CE was effective in maintaining catalase and peroxidase levels within the normal range and resisted the effects of lipid peroxidation of thiobarbituric acid reactive substances.

**Conclusion:**

In principle, the current study's findings scientifically validate the implication of *T. linearis* in metabolic syndrome and recommend further studies on molecular insights of the observed therapeutic activity.

## 1. Introduction

First coined in the 1980s as the “syndrome X,” metabolic syndrome (MetS) is now defined as an assembly of related metabolic abnormalities such as lipid metabolism (hypercholesterolemia and dyslipidemia), elevated blood pressure, disrupted glucose metabolism, and central obesity. Achievement of established threshold values for three of the five factors confers the presence of the syndrome [1]. Although NAFLD is not included in the diagnostic criteria of MetS, its association with manifestations of MetS is quite common. Considering obesity as the main contributor, NAFLD is thought to be the hepatic manifestation of metabolic syndrome [2].

Prevalence studies on metabolic syndrome in Asia Pacific's adult population conclude that urban Pakistan has the region's highest recorded prevalence (49%). The sudden and rapid shift in developing countries from high fiber, low fat, and low caloric diet to high total fats, red meats, and refined carbohydrates along with the unchecked consumption of inexpensive vegetable oils appears to be the prime role-playing factor in the development of conditions favourable for metabolic and cardiovascular irregularities [3].

Contrary to popular belief, adipose tissue cannot be merely treated as an energy reservoir, but it also serves a crucial function as an endocrine gland. It produces and releases a variety of biologically active substances known as adipocytokines, including “offensive” adipocytokines such as TNF *α*, interleukin 6, plasminogen activator inhibitor-1, angiotensinogen monocyte chemoattractant protein 1, and resistin and “defensive” adipocytokines such as adiponectin and leptin. The mechanism of pathogenesis of obesity-associated metabolic syndrome involves the imperfect regulation of these adipocytokines. Since metabolic syndrome is characterized by a changed oxidant/antioxidant balance, hence oxidative stress is strongly associated with metabolic syndrome and related complications. Despite advanced research and growing awareness in this field, current management strategies for obesity-associated metabolic syndrome fail to produce a sustained effect. Hence, being the established prime causative agent in the development of this condition, oxidative stress should be targeted for the development of new therapies [4].

*Thymus linearis* Benth. (family: Lamiaceae) (the plant name has been checked with http://www.theplantlist.org.) is a perennial plant found in the alpine areas of Pakistan, Nepal, India, and Afghanistan. It is commonly known as “Himalayan Thyme” in English. Other common names include “Creeping Thyme,” “Jangli Ajwain,” “Satar Farsi,” and “Tumuro.” Traditionally, it has been used for productive cough, high blood pressure, spasms, and headache as well as fever and inflammation [5]. It has also been used for the treatment of skin, eye, and liver diseases and has been also found to be used against hookworm [6]. This plant has also been found to have antibacterial, antiviral, and anticancer activities [5]. Furthermore, it has also been used as tonic, antiseptic, and for treating cold as well as herbal tea [7].

## 2. Material and Methods

### 2.1. Collection and Identification

Aerial parts of *T. linearis* were collected from Hunza, Gilgit Baltistan, Pakistan, in August 2017. Dr. Sher Wali Khan, Department of Botany, Karakoram International University, Gilgit Baltistan, identified the plant as *Thymus linearis* Benth. A voucher specimen with Accession # PHM 521 was registered in the herbarium of medicinal plants, Department of Pharmacy, Quaid-i-Azam University, Islamabad, Pakistan.

### 2.2. Extraction and Sample Preparation

Aerial parts of the plant were washed and shade dried at room temperature for a period of three weeks. The dried plant material was ground to form a fine powder (2500 g) which was then subjected to polarity-guided sonication-assisted maceration technique as described by Fatima et al. [8]. The solvents used were n-hexane, ethyl acetate, methanol, and distilled water. Powdered plant material was first soaked in n-hexane, for three days in 1000 ml Erlenmeyer flasks before being intermittently sonicated at 25 kHz. First, muslin cloth was used for filtering, and then, filter paper was used for fine filtration. The solvent was extracted from the filtrate using a rotary evaporator operating at 40°C under pressure. The same procedures were followed the second time using the same solvent. Similar procedure was modified for the remaining liquids, namely, EA, M, and DW. Crude plant extracts were kept in correctly labelled containers and kept in the refrigerator at 4°C after they had fully dried.

The five test samples included extracts obtained from n-hexane (NH), ethyl acetate (EA), methanol (M), and distilled water (DW), along with the combined mixture (CE) which was obtained by combining the four solvent extracts in a ratio equal to their extraction yield [8].

### 2.3. Standardization of Extracts

#### 2.3.1. Phytochemical Analysis

*(1) Total Phenolic Content (TPC) Estimation*. Test samples (20 *μ*l; 4 mg/ml DMSO) were mixed with the Folin-Ciocalteu (FC) reagent (90 *μ*l; 1 : 10 in distilled water), and sodium carbonate (90 *μ*l; 6% *w*/*v*) was added to the mixture. Absorbance of the reaction mixture was recorded using a microplate reader (Elx 800, Biotek USA) at 630 nm after incubation at 37°C for 30 minutes. Gallic acid and DMSO were used as positive and negative standards, respectively. TPC was then calculated using the linear equation of a standard curve prepared from absorbance of gallic acid at different concentrations (1.56, 3.12, 6.25, 12.5, and 25 *μ*g/ml). Resultant TPC was then expressed as *μ*g gallic acid equivalent (GAE) per mg extract [9].

*(2) Total Flavonoid Content (TFC) Estimation*. A mixture containing 20 *μ*l of test sample (4 mg/ml DMSO), 10 *μ*l of potassium acetate (1 M), 10 *μ*l of aluminium chloride (10% *w*/*v*), and 160 *μ*l distilled water was incubated for 30 min at room temperature (22°C). Absorbance was then measured. Calibration curve was drawn under the same experimental conditions using different concentrations of quercetin (2.5, 5, 10, 20, and 40 *μ*g/ml) as positive control and DMSO as negative control. Calculated TFC was expressed as *μ*g quercetin equivalent (QE) per mg extract [9].

*(3) Reverse Phase High-Performance Liquid Chromatography (RP-HPLC) Analysis*. The method and apparatus described by Fatima et al. [8] were employed with slight modifications. High-performance liquid chromatography was performed using Agilent Chem station Rev. B.02-01-SR1 (260) and Agilent 1200 series binary gradient pump coupled with diode array detector (DAD; Agilent Technologies, Germany). Zorbex-C8 analytical column (4.6 × 250 mm, 5 *μ*m particle size, Agilent, USA) was employed for reverse phase chromatographic analysis. Solvent A of the mobile phase was composed of acetonitrile-methanol–water-acetic acid in a ratio of 5 : 10 : 85 : 1, and solvent B consisted of acetonitrile-methanol-acetic acid in a ratio of 40 : 60 : 1. Solvent flow rate was set at 1 ml/min. Stock solutions of 18 phytochemical standards, i.e., vanillic acid, plumbagin, thymoquinone, gallic acid, catechin, syringic acid, coumaric acid, emodin, gentisic acid, caffeic acid, ferulic acid, luteolin, apigenin, myricetin, quercetin, and kaempferol, were prepared. The stock solutions prepared in methanol were then diluted to obtain final concentrations of 10, 20, 50, 100, and 200 *μ*g/ml of methanol. Before operation initiation, all mobile phase solutions, solutions of standards, and samples were degassed and filtered through a membrane filter (Millipore), 0.45 *μ*m in size. Ambient temperature was maintained for chromatographic operation. The column was reconditioned for 10 min in between analyses. The results were expressed as *μ*g/mg. UV-visible spectra and retention time obtained from the analysis of samples were compared with the results of standards for the identification and quantification of polyphenols.

#### 2.3.2. *In Vitro* Antioxidant Assays

*(1) Free Radical Scavenging Assay*. 2,2-Diphenyl-1-picrylhydrazyl- (DPPH-) based technique as described by Bibi et al. [10] was followed with minor modifications. The reaction mixture consisting of 10 *μ*l of test samples (4 mg/ml DMSO) and 190 *μ*l DPPH solution (9.6 mg/100 ml methanol) was incubated at 37°C in the dark for 60 min after which maximum absorbance was calculated at 515 nm. Ascorbic acid (1 mg/ml) was used as positive, and DMSO was used as negative control. Percent scavenging activity was calculated by the following formula:

(1) $$\begin{array}{c} \%\text{scavenging activity}=\frac{1-\text{absorbance of sample}}{\text{absorbance of negative control}}\times 100. \end{array}$$

Fifty percent inhibitory concentration (IC_50_) of samples exhibiting %scavenging ≥ 50 was evaluated using the dilution method at lower sample concentrations (200, 66.6, 22.22, and 7.4 *μ*g/ml).

*(2) Determination of Total Antioxidant Capacity (TAC)*. Phosphomolybdenum-based colorimetric assay was used for the estimation of total antioxidant capacity of extracts [11]. Test samples (100 *μ*l; 4 mg/ml DMSO) and 900 *μ*l TAC reagent (4 mM ammonium molybdate, 0.6 M sulphuric acid, and 28 mM sodium phosphate) were added to Eppendorf tubes and incubated at 95°C in a water bath for 90 min. After cooling, absorbance of the reaction mixture was measured at 630 nm. Ascorbic acid (100 *μ*l; 1 mg/ml) and DMSO were used as positive and negative controls, respectively. The assay was performed in triplicate. Calibration curve of ascorbic acid at final concentrations (100, 50, 25, 12.5, 6.25, and 3.12 *μ*g/ml) was drawn, and total antioxidant capacity was expressed as *μ*g ascorbic acid equivalent (AAE) per mg extract.

*(3) Determination of Total Reducing Power (TRP)*. Procedure based on the reduction of ferric chloride was followed as described earlier by Ahmed et al. [11]. Test sample (100 *μ*l; 4 mg/ml DMSO), 200 *μ*l of 0.2 M phosphate buffer (pH 6.6), and 250 *μ*l potassium ferric cyanide (1% *w*/*v*) were added to the Eppendorf tubes and incubated in a water bath at 50°C for 20 min, followed by the addition of 200 *μ*l of trichloroacetic acid (10% *w*/*v*). The mixture was centrifuged for 10 min at 3000 rpm. The supernatant (150 *μ*l) of each mixture was then transferred to the corresponding wells of a 96-well plate already containing 50 *μ*l of ferric cyanide (0.1% *w*/*v*) solution. Finally, absorbance was measured at 630 nm. The same procedure was followed for positive (gallic acid) and negative (DMSO) controls. Calibration curve of ascorbic acid at final concentrations (100, 50, 25, 12.5, 6.25, and 3.12 *μ*g/ml) was drawn, and total reducing power of the extracts was expressed as *μ*g ascorbic acid equivalent (AAE) per mg extract.

#### 2.3.3. *α*-Amylase Inhibition Assay

The inhibition of *α*-amylase activity was assessed according to a procedure previously followed by Zahra et al. [12]. Briefly, 10 *μ*l of sample solution (4 mg/ml) prepared in DMSO was added to wells of a 96-well plate along with 25 *μ*l of *α*-amylase stock solution (0.12 U/ml in 50 mM phosphate buffer, pH 6.8), 15 *μ*l phosphate buffer (50 mM; pH 6.8), and 40 *μ*l starch solution (2 mg/ml phosphate buffer). The plate was then incubated at 50°C for 30 minutes followed by the addition of 20 *μ*l of 1 M HCl and 90 *μ*l of iodine solution (5 mM iodine, 5 mM potassium iodide) for colour development. Positive and negative controls were prepared by replacing test samples with acarbose (250 *μ*M) and DMSO, respectively, in the designated wells. Blank solution was prepared by replacing the sample with equal quantity of phosphate buffer. Absorbance was measured at 540 nm using a UV-visible spectrophotometer (Elx 800, Biotek USA). The *α*-amylase inhibitory activity was calculated using the equation given below.

(2) $$\begin{array}{c} \%\text{inhibition}=\frac{\text{Abs}-\text{Abn}}{\text{Abb}-\text{Abn}}\times 100, \end{array}$$

where Abs is the absorbance of test sample, Abb is the absorbance of blank, and Abn is the absorbance of negative control.

Test samples exhibiting ≥50% inhibition were subjected to IC_50_ determination at 200, 66.6, 22.22, and 7.4 *μ*g/ml sample concentrations.

#### 2.3.4. Selection of Extract

The extracts subjected to *in vitro* testing showed a range of diverse results, such as DW extract showed highest phenolic content while EA extract showed highest flavonoid content and substantial alpha amylase inhibitory activity. M extract presented the highest antioxidant activity (TAC and TRP) while CE extract had noteworthy free radical scavenging activity. Therefore, CE extarct was selected for further *in vivo* testing. Moreover, the CE extract also exhibited excellent *in vitro* antioxidant and antidiabetic potential.

### 2.4. Animals and Modelling

A high-fat diet-induced obesity model as described by Fraulob et al. [13] was followed with some modifications. Twenty-eight male Sprague Dawley rats (~150-250 g) were selected for the experiment and maintained on 12 h light/dark cycle and at a temperature of 28°C. The animals were allowed free access to food and water. After one week of acclimatization, test animals were divided into 7 groups (*n* = 7 each). The distribution of groups is shown in Table 1.

Three groups were fed on standard laboratory diet (STD) and four on a high-fat diet (HFD). Standard laboratory diet was modified to include suet to prepare a high-fat diet. The HFD groups were also fed with 5 mg cholesterol/rat suspended in 10% *v*/*v* coconut oil for a period of 10 weeks. All the experiments were performed according to the guidelines of the ethical committee of QAU, ISB, Pakistan, for animal care. The experiments were approved under Letter # BEC-FBS-QAU2019-141. The study was planned according to the guidelines of the National Institute of Health (NIH), Islamabad, and minimal discomfort, pain, and distress to the test animal were ensured during the study.

### 2.5. Oral Glucose Tolerance Test

On the day of blood glucose measurement, food was removed, and the cage bedding was changed (to minimize coprophagy) 7–8 h before measurement [13]. Zero-minute glucose measurement was done by withdrawing blood from the rat's tail vein (3 *μ*l) and analysing it through glucometer (Accu-Chek Active, Roche, Germany). Glucose (25%, 2 ml) was administered orally to nonsedated animals. After glucose administration, glucose readings were again taken at 5, 15, 30, and 60 min.

### 2.6. Euthanasia

After 10-week period, the animals were anaesthetized with the help of chloroform, and blood samples were collected using cardiac puncture method. Plasma was isolated through centrifugation at 6000 rpm and 4°C for 15 minutes, and the samples were stored at -20°C until they were subjected to biochemical analysis.

The liver, fat pads (retroperitoneal and epididymis, mesenteric), kidneys, brain, and heart were isolated, washed with ice cold normal saline solution, and weighed. The weights of different groups were compared for analysis of the effect of high-fat diet and CE on organ weights. Liver sections were stored in 10% formalin for histopathological studies [13].

### 2.7. Determination of Homeostasis Model of Insulin Resistance (HOMA-IR)

On the final day of the experiment, after 7 hours of fasting, blood glucose and serum insulin were calculated using glucometer (Accu-Chek Active, Roche, Germany). Serum previously collected and stored was used for insulin estimation through ELISA kit (Elabscience Biotechnology Inc., USA). The calculated values were placed in the equation for HOMA-IR and thereby calculated [14].

(3) $$\begin{array}{c} \text{HOMA IR}=\frac{\text{serum insulin}\left(\text{mmol}/\text{l}\right)*\text{blood glucose}\left(\text{mmol}/\text{l}\right)}{22.5}. \end{array}$$

### 2.8. Histological Analysis

Liver and adipose pads were cut into small pieces and rinsed with saline. The pieces were then fixed with 10% formalin followed by cutting 25 *μ*m tissue sections. The sections were stained with eosin and haematoxylin. Fat sections were observed under light microscope (DIALUX 20 EB) at 40x, and images were photographed using HDCE-50B camera in order to assess adipocyte size which was later measured through ImageJ software. Liver sections were also observed for any signs of fat accumulation [13].

### 2.9. Serological Analysis

The high-density lipid profile (HDL-c) was estimated through colorimetric and precipitant method employing the Cholesterol HDL Precipitant enzymatic kit (Dialab, Austria). The cholesterol, total triglyceride content, and liver enzymes (ALT and AST) were assayed with the help of enzymatic colorimetric kits (Inoline, Merck, Pakistan). Low-density lipids (LDL-c) were calculated using the Friedewald formula [15].

(4) $$\begin{array}{c} \text{LDL}\left(\text{mg}/\text{dl}\right)=\frac{\text{total cholesterol}-\text{triglyceride}}{5-\text{HDL}}. \end{array}$$

### 2.10. Endogenous Antioxidant Assessment

#### 2.10.1. Catalase Activity Assessment

Catalase activity was analysed in serum according to the method adopted by Kazmi et al. [16]. The test mixture consisting of 250 *μ*l of phosphate buffer (50 mM, pH 5.0), 40 *μ*l of H_2_O_2_ (5.9 mM), and 10 *μ*l of the serum was analysed. Rate of change of absorbance per minute was observed at 240 nm using a microplate reader (Elx 800, Biotek USA) at 0, 1, and 2 min. Absorbance variation of 0.01 units per minute was regarded as one-unit catalase activity, and results were expressed as unit per min protein (U/min protein).

#### 2.10.2. Peroxidase (POD) Activity Assessment

The method adopted by Naz et al. [17] was employed for the assessment of peroxidase activity. The assay mixture consisted of 10 *μ*l of 20 mM guaiacol, 250 *μ*l of 50 mM phosphate buffer (pH 5.0), 30 *μ*l of 50 mM H_2_O_2_, and 10 *μ*l of serum. Absorbance of reaction mixture was recorded at 470 nm using a microplate reader (Elx 800, Biotek USA) after every minute for 2 min, and one-unit peroxidase activity was determined as an absorbance change of 0.01 units per minute. Results were calculated as unit per mg protein (U/mg protein).

#### 2.10.3. Superoxide Dismutase (SOD) Activity Assessment

According to the previously described method [17], the reaction mixture was prepared by mixing 11 *μ*l of phenazine methosulphate (186 *μ*M), 132 *μ*l of sodium pyrophosphate buffer (0.052 mM; pH 7.0), and 10 *μ*l of serum. The reaction was initiated by the incorporation of 22 *μ*l of 780 *μ*M NADH and stopped by the addition of 110 *μ*l glacial acetic acid. The absorbance of the reaction mixture was recorded at 560 nm, and the results were expressed as unit per mg protein (U/mg protein).

### 2.11. Assessment of Lipid Peroxidation

The protocol described by Sindhu et al. [18] was followed for assessment for lipid peroxidation in serum and tissue homogenates. Reaction mixture consisting of 20 *μ*l ferric chloride (100 mM), 200 *μ*l ascorbic acid (100 mM), 580 *μ*l phosphate buffer (0.1 M; pH 7.4), and 200 *μ*l of serum was incubated at 37°C for a period of 1 hr in water bath. The reaction was stopped by the incorporation of 1000 *μ*l of 10% trichloroacetic acid followed by 1000 *μ*l of 0.66% thiobarbituric acid. The mixture containing tubes were kept in boiling water for 20 min after which they were cooled on an ice bath and centrifuged at 2500 × g for 10 min. In order to quantify the amount of thiobarbituric acid reactive substances (TBARS) in samples, the absorbance of samples and reagent blank (containing all the reagents except test sample) was measured at 535 nm, and the results were expressed as nanomolar TBARS per min per mg protein (nM TBARS/min/mg protein).

### 2.12. Assessment of Genotoxicity

Genotoxic potential of the *T. linearis* extract was assessed through comet assay of brain, heart, liver, and kidney homogenates. The method established by Eid et al. [19] was followed, whereby methanol-dipped microscopic slides were burnt over flame in order to remove any mechanical oil or dust. The sterile plates were impregnated with 1% normal melting point agarose solution and were then allowed to cool off at 25°C. Small portions of fat pads were crushed into 1 ml cold lysing solution, and mixture of macerated tissue and 85 *μ*l of low melting point agarose (LMPA) was spread over the coated slides. The slides were immediately covered with coverslips and placed on ice packs for solidification. A layer of 80 *μ*l LMPA was added to slides prior to the second round of solidification. After the removal of cover slips, plates were prepared for electrophoresis by placing them in cold lysing solution for 2 hr at 4°C. Electrophoresis was conducted in an electrophoresis tank filled with electrophoresis buffer. The process was performed at 25 V for 20 min after which the slides were twice impregnated with neutralization buffer. The slides were then stained with ethidium bromide and examined under an electron microscope. DNA damage evaluation was done with the help of CASP 1.2.3.b software.

### 2.13. Statistical Analysis

The data obtained was presented in the form of mean ± standard deviation. The data were further analysed statistically using one-way ANOVA followed by Tukey's multiple comparison test. The statistical significance was set at ˂0.05. Graphs were designed with the help of GraphPad 5.

## 3. Results and Discussion

The herbal tea of *T. linearis* is extensively used in traditional medicine for diabetes and obesity; however, there was no scientific evidence hitherto. Therefore, the present study, after scientifically proving the plant's effectiveness against metabolic syndrome, validates the ethnopharmacological use of *Tumuro* tea for obesity and diabetes.

This study presents the first comprehensive investigation of therapeutic potential of *T. linearis*' extract against different aspects of metabolic syndrome. It establishes that the extract can effectively reduce weight gain in rats fed on high-fat diet. The extract has also proven successful in regulating lipid metabolism and glucose and insulin intolerance. Experiments on liver enzymes reveal that said extract maintains liver enzymes within the normal range even when the administration of high-fat diet raises the levels in the control group. Also, a previous study has shown that the plant's aerial parts possess antihypertensive properties [20]. Owing to these attributes, *T. linearis* extracts can be envisaged as a valuable herbal remedy against metabolic syndrome.

### 3.1. Percentage Extract Yield

Percentage extract yield assessment showed that the highest amount of phytochemicals was extracted in DW extract (Table 2). The four extracts were mixed in a ratio (0.39 : 1.28 : 1.57 : 6.74) corresponding to the % extract yield of the respective extract in order to form a mixture or combined extract (CE).

### 3.2. Phytochemical Analysis

**Figure 1:**
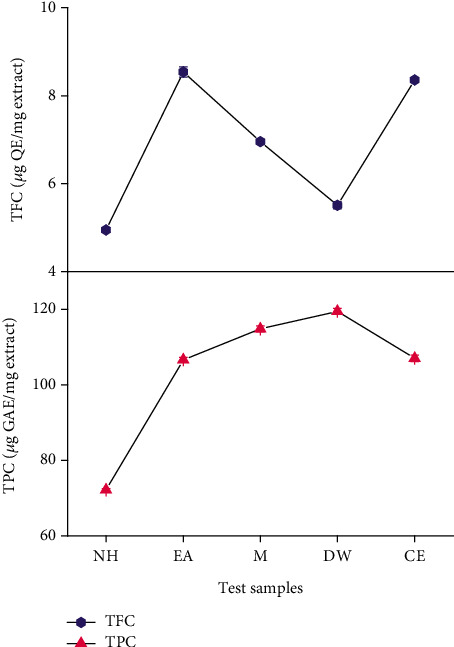
Graphical representation of TPC and TFC estimation of *T. linearis* extracts. Note: values are expressed as mean of triplicate ± standard deviation. TPC: total phenolic content; TFC: total flavonoid content.

Phytochemical analysis of plant extracts shows that the plant is richly composed of phenolics and flavonoids. The highest concentration of total phenolic contents equivalent to gallic acid was found in 119.4 ± 0.82 *μ*g/mg of DW extract. The highest flavonoid concentration equivalent to quercetin was present in 8.53 ± 0.11 *μ*g/mg of EA (Figure 1).

**Figure 2:**
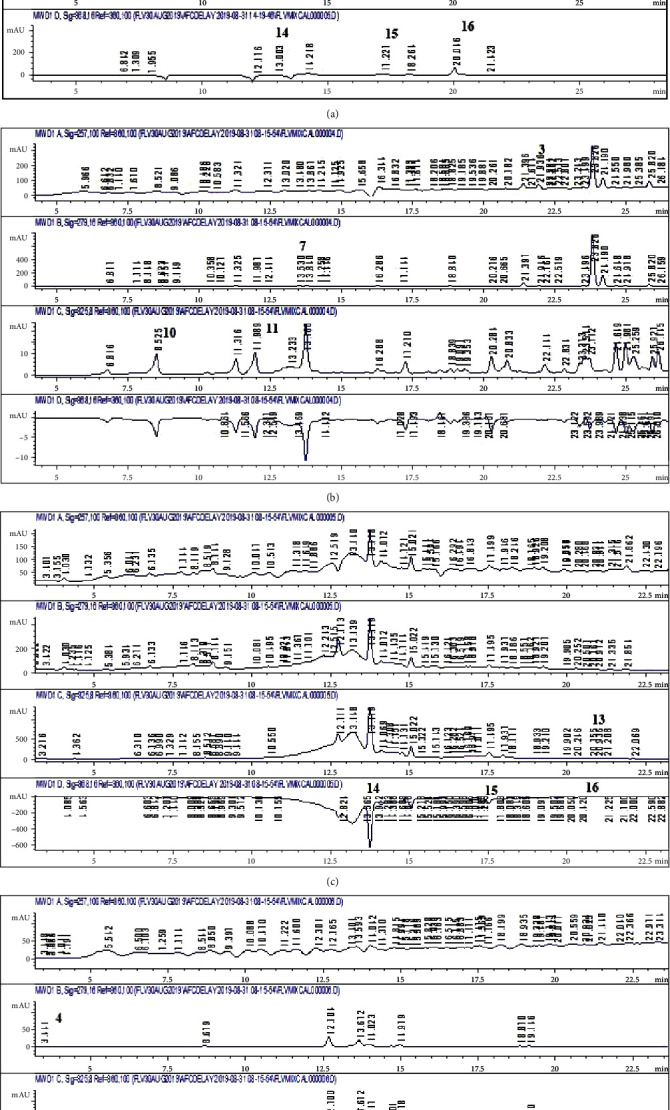
(a) HPLC chromatogram of standard polyphenols. (b) HPLC chromatogram of ethyl acetate extract. (c) HPLC chromatogram of methanol extracts. (d) HPLC chromatogram of distilled water extract. Note: 1, vanillic acid; 2, plumbagin; 3, thymoquinone; 4, gallic acid; 5, catechin; 6, syringic acid; 7, coumaric acid; 8, emodin; 9, gentisic acid; 10, caffeic acid; 11, ferulic acid; 12, luteolin; 13, apigenin; 14, myricetin; 15, quercetin; 16, kaempferol.

Detailed RP-HPLC, performed for the first time on *T. linearis* extracts, reveals that the most prominent phytoconstituents are flavonoids like myricetin, kaempferol, and apigenin (Figures 2(a)–2(d)). The most noteworthy phenolic is gallic acid with a concentration of 0.18 *μ*g/mg in CE extract and myricetin in M extract (1.43 *μ*g/mg) (Table 3). Phenolics and flavonoids, through numerous mechanisms, treat different physiological dysregulations. This attribute is later associated in this study with the therapeutic properties of the plant.

### 3.3. Antioxidant Potential

**Figure 3:**
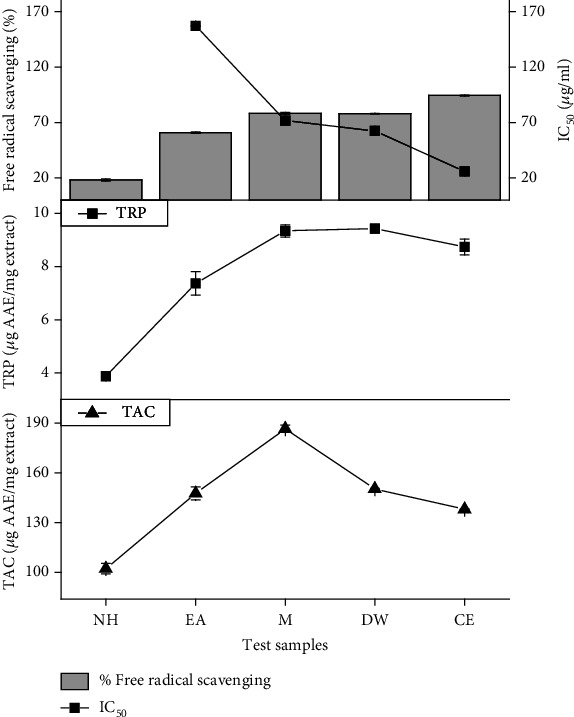
Graphical representation of DPPH free radical scavenging activity, IC_50_ estimation, TAC, and TRP analysis of *T. linearis* extracts. Note: values are presented as average of triplicate ± standard error. TAC: total antioxidant capacity; TRP: total reducing power.

The plant extracts have shown remarkable antioxidant potential in *in vitro* assays. The plant showed excellent activity in FRSA assay, with %scavenging as high as 94.7% (CE extract) (Figure 3). This result is in complete agreement with previous antioxidant studies [21] performed on the plant, where aqueous extract showed 95% scavenging at 100 *μ*g/ml. In addition, prominent activity was shown by M and DW in TAC and TRP assays (Figure 3). The phytochemicals detected in plant extracts have previously shown to exhibit excellent antioxidant properties. Myricetin and kaempferol possess excellent ability to scavenge DPPH (2,2-diphenyl-1-picrylhydrazyl) radicals. The outstanding DPPH scavenging ability of *T. linearis* extracts could be due to presence of these phytoconstituents [22].

### 3.4. *α*-Amylase Inhibition Activity

**Figure 4:**
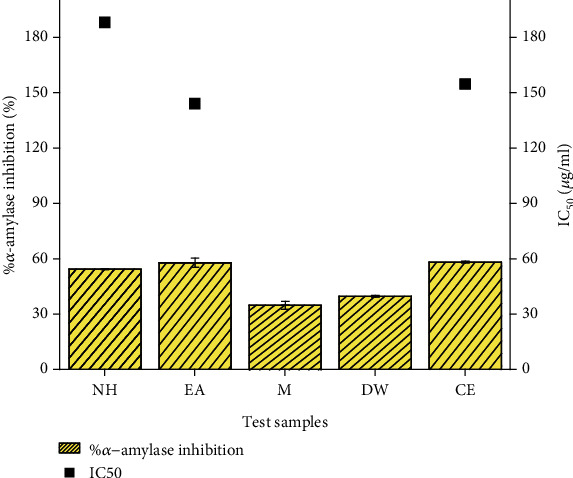
Graphical representation of *α*-amylase inhibition and IC_50_ values of *T. linearis* extracts. Note: acarbose is used as a standard with an IC_50_ 33.73 *μ*g/ml. Values are presented as mean ± standard deviation.

This study for the first time investigates the effect of *T. linearis* on glucose-metabolizing enzyme, *α*-amylase. It was found that the EA and CE extracts inhibit *α*-amylase enzyme (Figure 4) and hence contribute to reduction in postprandial hyperglycemia. This effect can also be linked with the presence of myricetin which has shown significant *α*-amylase inhibition activity in previous studies [23].

The *in vitro* studies were helpful in selecting an extract for *in vivo* testing. Polarity-guided successive extraction produced four different polarity extracts. These extracts showed diverse results in *in vitro* studies. Hence, it was imperative to prepare an extract which possessed all those properties exhibited by individual extracts. Keeping this in mind, the combined extract containing the four different extracts in a ratio equal to their extraction efficiency was prepared. The CE itself showed significant results in all *in vitro* tests.

### 3.5. Effect on Obesity

**Figure 5:**
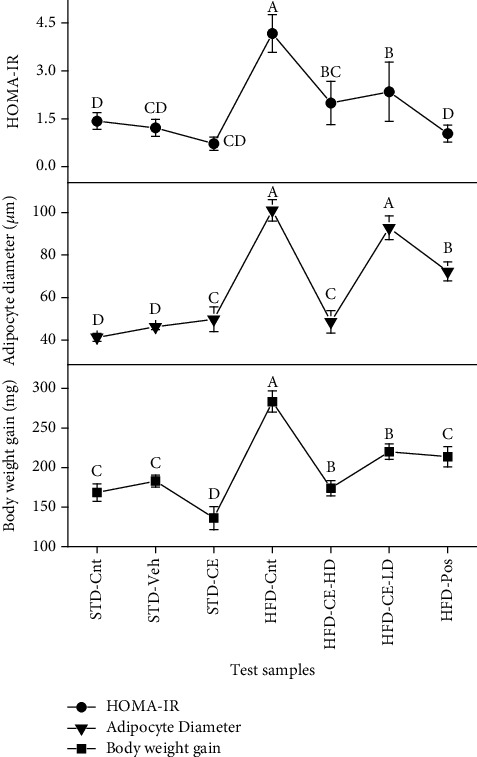
Effect of standard diet, high-fat diet, and *T. linearis* extract on body weight gain and adipocyte diameter and HOMA-IR over period of 10 weeks. Note: means with different superscript (A–D) letters in the column are significantly different from one another according to Tukey's multiple comparison at *p* < 0.05. HOMA-IR: determination of homeostasis model of insulin resistance.

**Figure 6:**
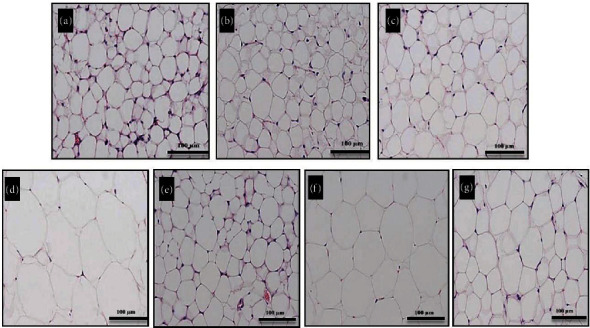
Pictorial representation of adipose tissue: (a) STD-Cnt, (b) STD-Veh, (c) STD-CE, (d) HFD-Cnt, (e) HFD-CE-HD, (f) HFD-CE-LD, and (g) HFD-Pos. Note: STD: standard laboratory diet-control; Cnt: control; Veh: vehicle; CE: combined extract; HFD: high-fat diet; HD: high dose; LD: low dose; Pos: positive.

The first most important aspect of metabolic syndrome is obesity. Three different parameters, i.e., amount of weight gained, organ weight, and diameter of adipocytes, were assessed to analyse the effect of plant extract on obesity. Different groups were simultaneously fed on normal diet, high-fat diet, and the plant extract. There was no significant difference in the animal weights up to 5 weeks, after which the animals fed on high-fat diet started gaining weight. By the end of the 10^th^ week, the high-fat diet only group showed a significant increase (*p*˂0.05) in weight as compared to the extract-administered groups which exhibited a prominent reduction in weight gain (Figure 4). It was observed that this resistance changed in a dose-dependent manner. The extract resisted significant weight gain despite administration of a high-fat diet. The antiobesity property of plant extract decreased in a dose-dependent manner, and the activity of the high dose was comparable with the marketed antiobesity drug, orlistat. Moreover, the extract was also able to reduce weight of animals fed on standard diet only. The compared organ weights also showed that CE was able to check weight gain in liver, epididymal, retroperitoneal, and mesenteric fat pads as compared to the organs (liver, epididymal, and retroperitoneal fat pads) of rats fed on high-fat diet only. No significant differences were seen in brain, heart, and kidney weights (Table 4). Moreover, adipocyte hypertrophy, an indicator of obesity, was also studied. Adipose cells store lipids, and upon excess of lipid accumulation, the adipose cells increase in size. This results in an inflamed adipose tissue which consequently leads to development of insulin resistance and T2DM [24]. Our study shows that high-fat diet induced significant (*p*˂0.05) adipocyte hypertrophy, but the adipocytes of animals receiving oral *T. linearis* extract (100 mg/0.75% CMC solution) did not develop hypertrophy and hence did not develop obesity despite administration of high cholesterol diet (Figures 5 and 6).

Studies on myricetin reveals that it has significant antiobesity properties. In one particular *in vivo* study conducted on rats, myricetin accelerated the process of fatty acid oxidation in rat liver, thereby reducing body weight and fat accumulation [22]. Moreover, another study conducted on mouse preadipocyte 3T3-L1 cells highlights the antiadipogenic activity of flavonols like kaempferol and myricetin. The flavonoids decrease lipid accumulation in adipocytes. They achieve this by inhibiting the differentiation of preadipocytes to mature adipocytes. This results in decrease in lipid accumulation. This disruption of differentiation is confirmed and associated with the downregulation of CEBPA, PPAR*γ*, and FABP4 genes which are connected with adipocyte differentiation [22]. Another study has shown that flavone, apigenin, presents similar effects on mouse 3T3-L1 cells through activation of AMPK pathway and decreased expression of adipogenic genes, thereby decreasing weight gain and reducing body fat content [25]. Moreover, ferulic acid has shown antiobesity properties similar to marketed drug, sibutramine. This flavonoid reduced body weight and inhibited visceral fat accumulation when administered orally to Swiss mice along with a high-fat diet by lowering plasma leptin and ghrelin levels. The antiobesity activity of *T. linearis* CE could also be attributed to the presence of gallic acid which promotes *in vitro* inhibition of pancreatic lipase and *in vivo* suppression of weight gain [26].

### 3.6. Effect on Hyperglycemia and Insulin Resistance

The second part of our study focused on the effect of *T. linearis* on insulin resistance. Insulin resistance can be considered the backbone which links obesity, diabetes, and liver conditions. HOMA-IR is a convenient method for assessment of insulin resistance. Undertaken study showed that 100 mg of CE extract significantly (*p*˂0.05) subdued the development of insulin resistance—as evident by HOMA-IR values—as compared to the control group. The high dose had even better insulin modulatory effects than the marketed drug, orlistat.

**Figure 7:**
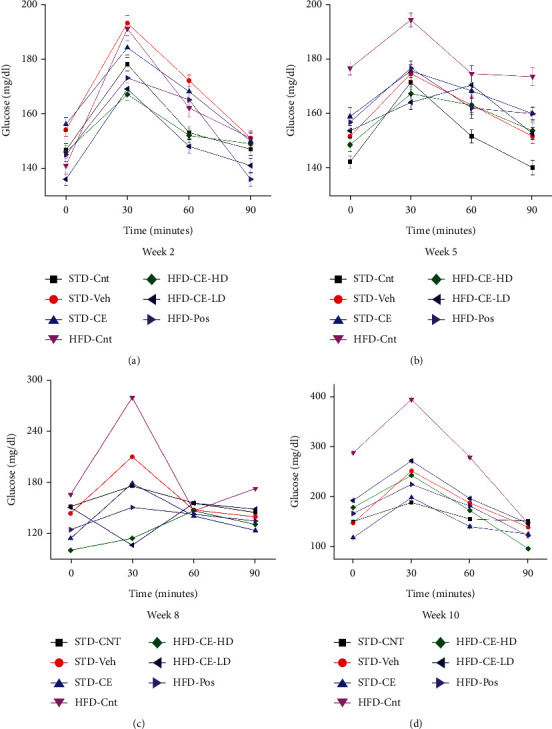
Glucose tolerance results of rats administered 25% glucose solution (400 mg/2 ml).

Glucose metabolism achieved by insulin can be seriously affected in metabolic conditions, wherein body cells grow resistant to metabolic effects of insulin. A deranged glucose metabolism is a direct precursor of type 2 diabetes [27]. The glucose tolerance assay showed that glucose tolerance decreased with feeding on high-fat diet, and therefore, the rats included in HFD-Cnt became glucose intolerant with time as assessed by the time taken for blood glucose levels to reach normal levels after glucose administration. The rats taking plant extract resisted the development of glucose intolerance even after administration of high-fat diet. The groups on standard diet did not show any difference in glucose tolerance (Figures 7(a)–7(d)).

Myricetin, one of the flavonoids detected in *T. linearis* extracts, has shown remarkable improvement in insulin resistance. It achieves this by increasing the expression of GLUT4 and augmenting the phosphorylation of Akt (also known as protein kinase B) and insulin receptor substrate 1 (IRS1). Likewise, kaempferol also acts in a similar fashion and decreases hyperglycemia and enhances glucose uptake in cells through increased expression of Akt, augmented cAMP, and enhanced synthesis of insulin in islet cells. Kaempferol has also exhibited stimulation of glucose uptake through protein kinase C and PI3K pathways [26]. Another flavonoid, apigenin, has also gained reputation for improving insulin resistance and hyperinsulinemia. It achieves this by inhibiting hepatic enzymes involved in gluconeogenesis, and this inhibition leads to reduction in insulin resistance [28]. Moreover, ferulic acid, owing to its potent antioxidant activities, is used for its synergistic effects with other antidiabetic drugs [28].

### 3.7. Effect on Lipid Metabolism

Pathological alterations in lipid metabolism (dyslipidemia and hypercholesterolemia) are important manifestations of metabolic syndrome [29]. *T. linearis* CE extract, even upon administration of high-fat diet, significantly (*p* < 0.05) resisted increase in triglyceride and cholesterol levels as compared to the high-fat diet, low-dose, and standard drug fed groups. The extract also caused an increase in high-density lipid levels (Table 5). This is in agreement with a previous study conducted on *T. linearis* (Aktar et al., 2016). However, contrary to the previous study, the current study showed a reduction in triglyceride levels but no significant effect on LDL-C levels. Moreover, the lipid-lowering effect of study plant was more pronounced in groups fed on high-fat diet than in groups fed on standard laboratory diet.

Previous studies show that gallic acid, a phenol detected in the plant extract, has established activity in reducing hyperlipidemia with marked decrease in low-density lipids and triglycerides and increase in high-density lipids [30]. This is the same pattern as observed in our study. Moreover, myricetin has proven effective in improving hypercholesterolemia and hypertriglyceridemia. Induction of hepatic PPAR*γ* expression and reduction in the expression of SREBP are the plasma lipid-regulating mechanisms of myricetin [31]. Kaempferol through the same mechanism reduces plasma triglyceride levels [32].

### 3.8. Effect on Nonalcoholic Fatty Liver Disease

**Figure 8:**
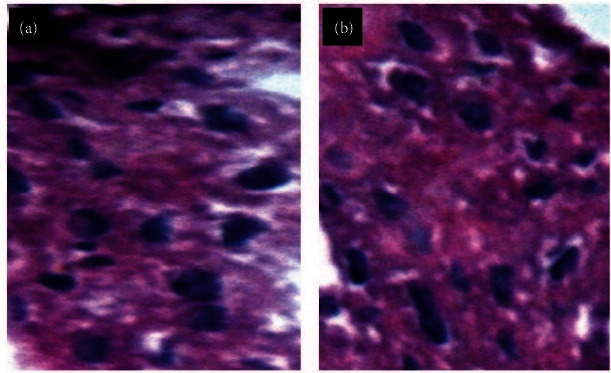
Pictorial representation of hepatic histology of (a) STD-Cnt and (b) HFD-Cnt. Note: STD: standard laboratory diet-control; Cnt: control; Veh: vehicle; CE: combined extract; HFD: high-fat diet; HD: high dose; LD: low dose; Pos: positive.

NAFLD is described as increased fatty aggregation in the liver, increasing 5 to 10% more than the usual weight. The undertaken study is aimed at establishing the effect of *T. linearis* on NAFLD through study of histological liver slides. However, the slides could not show suitable development of fatty liver in control as well (Figure 8). Hence, we lack the evidence to establish extract as either effective or ineffective against fatty acid infiltration in the liver. The understood cause could be that the study did not continue for a prolonged period of time, and hence, development of a fatty liver could not be achieved. Another important aspect of NAFLD is altered liver enzymes. Alterations in liver enzymes are indicative of hepatic damage. Liver enzyme levels when assessed in current study showed elevation in the control group fed on high-fat diet, while they remained controlled in the extract-administered groups (Table 6).

Studies reveal that myricetin could prove effective in treating hepatic steatosis since it normalizes liver enzymes and prevents hepatic accumulation of lipids by increasing the translocation of hepatic nuclear Nrf2, enhanced expression of heme oxygenase-1 (HO-1), and NAD(P)H quinone dehydrogenase 1 (NQO1) [33]. Kaempferol induces hepatic autophagy through activation of PPAR*α* and PPAR*δ*, and this reduces lipid droplet accumulation in the liver [34].

### 3.9. Effect on Endogenous Antioxidants and Lipid Peroxidation

**Figure 9:**
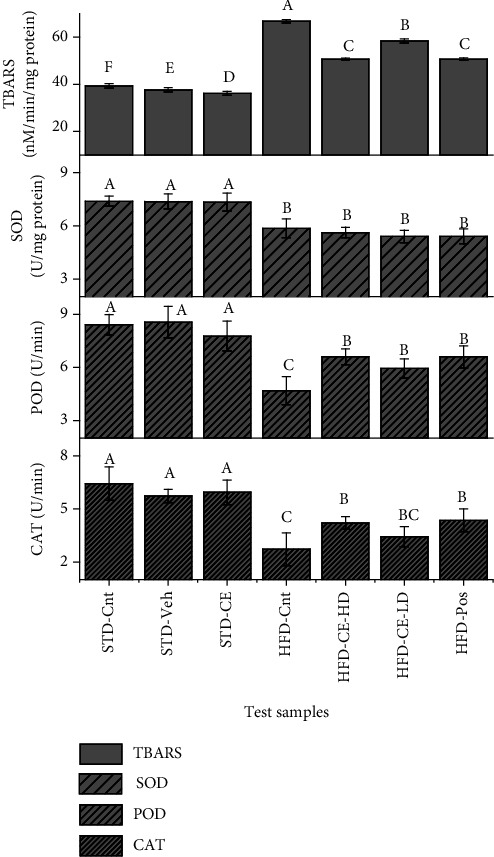
Graphical representation of effects of *T. linearis* extracts, standard diet, high-fat diet, and positive control on CAT, POD, SOD, and TBARS values. Note: means with different superscript (A–F) letters in the column are significantly different from one another according to Tukey's multiple comparison at *p* < 0.05. CAT: catalase; POD: peroxidase; SOD: superoxide dismutase; TBARS: thiobarbituric acid reactive substances.

Endogenous antioxidants were also assessed in our study in order to establish a better correlation of *T. linearis*' effects with its antioxidant potential. The endogenous antioxidants (CAT and POD) were significantly decreased (*p*˂0.05) in the HFD-Cnt group as compared to the HFD-CE-HD group, but there was no prominent effect on SOD values (Figure 8). Moreover, high-fat diet caused a significant (*p*˂0.05) lipid peroxidation—as evident by TBARS values—while high dose of the plant extract resisted the anomaly. The low dose and positive control failed to show any significant change in TBARS concentration (Figure 9).

Previous studies reveal that myricetin is effective in restoring the activity of endogenous antioxidant, catalase, but failed to show significant SOD-restoring ability [35]. This correlates with the activity shown by our study plant. Kaempferol also possesses strong potential to recondition endogenous antioxidants and oxidative stress-induced lipid peroxidation [36]. Similar results were achieved when rat hepatic preparations were treated with myricetin [37].

### 3.10. Assessment of Genetic Safety

**Figure 10:**
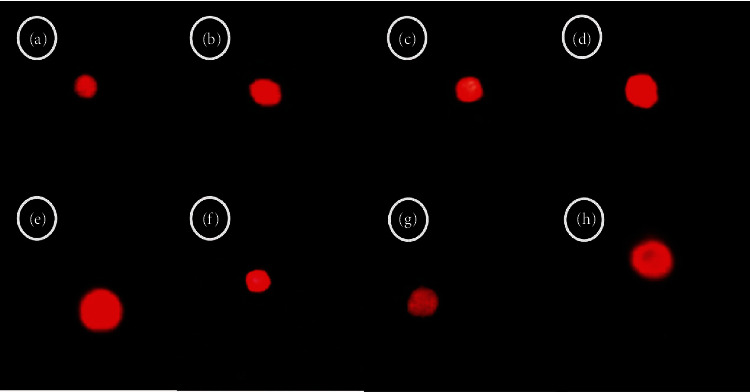
Fluorescence photomicrograph of effects of *T. linearis* extracts on DNA integrity. (a) STD-Cnt-brain, (b) STD-Cnt-heart, (c) STD-Cnt-liver, (d) STD-Cnt-kidney, (e) STD-CE-brain, (f) STD-CE-heart, (g) STD-CE-liver, and (h) STD-CE-kidney. Note: STD: standard laboratory diet-control; Cnt: control; Veh: vehicle; CE: combined extract.

The effect of the plant extract on genetic material was also observed. As compared to the standard control, we concluded that the plant shows nonsignificant tail DNA migration, and hence, it is not harmful to rat genetic material (Figure 10 and Table 7). This study presents the first report of genetic safety of *T. linearis* Benth. extracts.

The mechanisms through which extract of *T. linearis*. Benth in Wall resists development of obesity, diabetes, and nonalcoholic fatty liver disease may be related to the presence of various phytochemicals which are effective against these conditions. These flavonoids work through various pathways, and the mechanism of action of study plant's extracts may be one of these pathways. Another possible mechanism could be the action of extracts against oxidative stress induced through high-fat diet. As mentioned in Introduction, the pathophysiology of metabolic syndrome involves formation of different free radicals which further disrupt the release of adipocytokines. It is possible that these extracts work through the oxidative pathway to alleviate different aspects of metabolic syndrome. Further study on mechanism is required for more insight on this subject.

## 4. Conclusion

*Thymus linearis* Benth in Wall also known as Himalayan Thyme owing to its traditional use was appraised for its potential against metabolic syndrome. Noteworthy activities in vitro as well as remarkable weight reduction, glucose and lipid metabolism stabilisation, and hepatoprotective attributes against influences of a fatty diet prove that *Thymus linearis* can be a noteworthy lead as a drug for metabolic syndrome. These significant properties could be attributed to the exceptional presence of phytochemicals quantified in HPLC analysis, antioxidants, and the excellent ability to restore endogenous antioxidants in Sprague Dawley rats.

## Figures and Tables

**Table 1:** Distribution of animals according to groups.

Group number	Group name	Group code
Standard diet fed groups		
Group I	Control	STD-Cnt
Group II	Vehicle	STD-Veh
Group III	Extract 100 mg/kg in CMC (1 ml; 0.75% in DW)	STD-CE

High-fat diet fed groups		
Group IV	Control	HFD-Cnt
Group V	Extract 100 mg/kg in CMC (1 ml; 0.75% in DW)	HFD-CE-HD
Group VI	Extract 50 mg/kg in CMC (0.5 ml; 0.75% in DW)	HFD-CE-LD
Group VII	Orlistat 2 mg/kg in CMC (0.5 ml; 0.75% in DW)	HFD-Pos

STD: standard laboratory diet-control; Cnt: control; Veh: vehicle; CE: combined extract; HFD: high-fat diet; HD: high dose; LD: low dose; Pos: positive.

**Table 2:** Percent extract recovery of different *Thymus linearis* extracts.

Sr. no.	Solvent	Approximate polarity index	%*w*/*w* extract yield
1.	n-Hexane	0.1	0.68
2.	Ethyl acetate	4.4	2.29
3.	Methanol	5.1	2.79
4.	Distilled water	10.2	11.2

**Table 3:** Phenolic composition (*μ*g/mg extract) of different *Thymus linearis* extracts.

Sr. no	Standard polyphenols	Concentrations (*μ*g/mg)				
		Rt (nm)	EA	M	DW	CE
1.	Thymoquinone	257	0.18			0.023
2.	Gallic acid	279		0.3	0.21	0.18
3.	p-Coumaric acid	279	0.16			0.2
4.	Caffeic acid	325	0.31			0.039
5.	Ferulic acid	325	0.46			0.058
6.	Apigenin	325		0.5		0.078
7.	Myricetin	368		1.43		0.22
8.	Kaempferol	368		0.71		0.11

Note: EA: ethyl acetate extract; M: methanol extract; DW: distilled water extract; CE: combined extract; Rt: retention times.

**Table 4:** Effect of *Thymus linearis* extracts on organ weight.

Groups	Liver (g)	Epidydimal fat pads (g)	Retroperitoneal fat pads (g)	Mesenteric fat (g)	Kidneys (g)	Heart (g)	Brain (g)
STD-Cnt	10.58 ± 0.75^c^	4.01 ± 0.36^c^	8.00 ± 0.60^c^	2.11 ± 0.24^c^	2.00 ± 0.40^a^	0.73 ± 0.19^ab^	1.55 ± 0.31^ab^
STD-Veh	9.14 ± 0.82^cd^	4.12 ± 0.53^bc^	9.4 ± 0.82^b^	3.30 ± 0.91^bc^	1.96 ± .054^ab^	0.65 ± 0.22^b^	1.36 ± 0.11^ab^
STD-CE	9.95 ± 0.45^cd^	4.39 ± 0.67^bc^	3.94 ± 0.53^d^	2.61 ± 0.04^c^	2.06 ± 0.64^a^	0.59 ± 0.07^b^	1.39 ± 0.25^ab^
HFD-Cnt	16.85 ± 0.91^a^	7.41 ± 0.55^a^	11.99 ± 0.72^a^	5.81 ± 0.46^a^	2.05 ± .23^a^	0.89 ± 0.39^ab^	1.41 ± 0.24^ab^
HFD-CE-HD	13.21 ± 0.85^b^	4.47 ± 0.62^b^	9.56 ± 0.92^b^	3.05 ± 0.66^b^	2.04 ± 0.44^a^	1.05 ± 0.11^a^	1.60 ± 0.35^a^
HFD-CE-LD	13.69 ± 0.70^b^	6.21 ± 0.40^ab^	11.00 ± 0.69^ab^	3.3 ± 0.62^bc^	2.00 ± 0.59^a^	0.79 ± 0.42^ab^	1.77 ± 0.13^a^
HFD-Pos	11.75 ± 0.97^c^	4.03 ± 0.49^c^	8.8 ± 0.92^c^	3.15 ± 0.55^b^	2.29 ± 0.55^ab^	0.84 ± 0.51^ab^	1.59 ± 0.16^a^

Note: results are presented as mean ± SD (*n* = 4). Means with different superscript (a-d) letters in the column are significantly different from one another according to Tukey's multiple comparison at *p* < 0.05. STD: standard laboratory diet-control; Cnt: control; Veh: vehicle; CE: combined extract; HFD: high-fat diet; HD: high dose; LD: low dose; Pos: positive.

**Table 5:** Effect of *Thymus linearis* extract, standard, and high-fat diet on lipid profile.

Groups	Cholesterol (mg/dl)	Triglycerides (mg/dl)	HDL-C (mg/dl)	LDL-C (mg/dl)
STD-Cnt	176.01 ± 6.59^d^	157.9 ± 7.64^e^	77.5 ± 4.5^a^	126.3 ± 5.04^b^
STD-Veh	196.1 ± 8.77^c^	145.4 ± 7.14^e^	37.4 ± 3.63^e^	107.8 ± 7.83^c^
STD-CE	145.08 ± 9.56^e^	151.8 ± 7.53^e^	53.2 ± 4.82^cd^	108.2 ± 7.28^c^
HFD-Cnt	272.8 ± 7.75^a^	259.8 ± 8.2^a^	49.6 ± 4.41^d^	153.0 ± 6.7^a^
HFD-CE-HD	199.4 ± 8.34^c^	220.2 ± 10.17^c^	66.9 ± 4.47^ab^	117.6 ± 8.95^bc^
HFD-CE-LD	217.7 ± 8.06^b^	245.7 ± 9.08^b^	63.7 ± 2.36^bc^	143.3 ± 6.44^a^
HFD-Pos	119.0 ± 8.06^f^	173.8 ± 8.12^d^	52.3 ± 4.16^cd^	127.9 ± 5.73^b^

Note: results are presented as mean ± SD (*n* = 4). Means with different superscript (a-e) letters in the column are significantly different from one another according to Tukey's multiple comparison at *p* < 0.05. STD: standard laboratory diet-control; Cnt: control; Veh: vehicle; CE: combined extract; HFD: high-fat diet; HD: high dose; LD: low dose; Pos: positive.

**Table 6:** Effects of *Thymus linearis* extracts, standard, and high-fat diet on liver profile.

Groups	ALT (U/l)	AST (U/l)
STD-Cnt	33.7 ± 4.3^cd^	37.8 ± 3.3^d^
STD-Veh	31 ± 4.0^d^	29.1 ± 5.4^e^
STD-CE	32.4 ± 4.4^cd^	34.1 ± 2.4^de^
HFD-Cnt	63 ± 6.3^a^	80.1 ± 4.7^a^
HFD-CE-HD	39.2 ± 4.6^bc^	31.0 ± 3.2^c^
HFD-CE-LD	42 ± 2.9^b^	60.0 ± 2.7^b^
HFD-Pos	33.1 ± 4.3^cd^	45.1 ± 3.5^c^

Note: results are presented as mean ± SD (*n* = 4). Means with different superscript (a-e) letters in the column are significantly different from one another according to Tukey's multiple comparison at *p* < 0.05. STD: standard laboratory diet-control; Cnt: control; Veh: vehicle; CE: combined extract; HFD: high-fat diet; HD: high dose; LD: low dose; Pos: positive.

**Table 7:** Effects of *Thymus linearis* extracts on DNA integrity in brain, heart, liver, and kidney homogenates.

Groups	Comet length (*μ*m)	Head length (*μ*m)	Tail length (*μ*m)	%DNA in head	%DNA in tail
Brain					
STD-Cnt	34 ± 2.5^a^	31 ± 1.4^a^	3 ± 0.1^e^	99.9 ± 0.1^a^	0.003 ± 0.01^a^
STD-CE	32 ± 1.5^a^	30.5 ± 3.14^a^	2 ± 0.8^e^	99.8 ± 2.0^a^	0.13 ± 1.8^b^

Heart					
STD-Cnt	36 ± 2.0^a^	33 ± 0.9^a^	3 ± 1.9^a^	98.7 ± 0.4^a^	1.28 ± 0.1^a^
STD-CE	36 ± 3.6^a^	33 ± 1.7^a^	3 ± 0.8^a^	100.0 ± 0.6^a^	−0.01 ± 0.8^a^

Liver					
STD-Cnt	32 ± 1.8^a^	29 ± 1.7^a^	3 ± 1.3^a^	97.3 ± 0.4^a^	2.6 ± 0.12^a^
STD-CE	36 ± 2.3^a^	33 ± 2.0^a^	3 ± 1.8^a^	98.8 ± 0.8^a^	0.2 ± 0.26^a^

Kidneys					
STD-Cnt	30 ± 2.6^a^	27 ± 0.8^a^	3 ± 0.9^a^	99.9 ± 0.5^a^	0.006 ± 0.21^a^
STD-CE	28 ± 1.7^a^	25 ± 0.9^a^	3 ± 2.8^a^	100.0 ± 0.2^a^	−0.03 ± 0.16^a^

Note: results are presented as mean ± SD (*n* = 4). Means with different superscript (a-e) letters in the column are significantly different from one another according to Tukey's multiple comparison at *p* < 0.05. STD: standard laboratory diet-control; Cnt: control; CE: combined extract.

## Data Availability

All data is included.

## Abbreviations

AMPK: Adenosine monophosphate-activated protein kinase
cAMP: Cyclic adenosine monophosphate
CE: Combined extract
CMC: Carboxymethyl cellulose
DPPH: 2,2-Diphenyl-1-picrylhydrazyl
DW: Distilled water
EA: Ethyl acetate
FABP: Fatty acid-binding protein
FRSA: Free radical scavenging assay
GLUT4: Glucose transporter type 4
HDL-c: High-density lipid
HFD: High-fat diet
HFD-CE-HD: High-fat diet+high dose of cumulative extract
HFD-CE-LD: High-fat diet+low dose of cumulative extract
HFD-Cnt: High-fat diet+control
HFD-Pos: High-fat diet+positive
HO-1: Heme oxygenase-1
HOMA-IR: Homeostasis model of insulin resistance
ISB: Islamabad
LDL-C: Low-density lipid
M: Methanol
MetS: Metabolic syndrome
NADPH: Nicotinamide adenine dinucleotide phosphate
NAFLD: Nonalcoholic fatty liver disease
NH: n-Hexane
NIH: National Institute of Health
PI3K: Phosphoinositide 3-kinases
POD: Peroxidase
PPAR*γ*: Peroxisome proliferator-activated receptor gamma
QAU: Quaid-i-Azam University
RP-HPLC: Reverse phase high-performance liquid chromatography
SOD: Superoxide dismutase
SREBP: Sterol regulatory-element binding proteins
STD: Standard laboratory diet
STD-CE: Standard diet+cumulative extract
STD-Cnt: Standard diet+control
STD-Veh: Standard diet+vehicle
T. linearis: Thymus linearis
T2DM: Type 2 diabetes mellitus
TAC: Total antioxidant capacity
TBARS: Thiobarbituric acid reactive substances
TRP: Total reducing power.
